# Association Between Alcohol Use Patterns and Insomnia Symptoms or Poor Sleep Quality Among Adult Women: An Internet Cross-Sectional Survey in Japan

**DOI:** 10.3390/clockssleep7010005

**Published:** 2025-02-13

**Authors:** Suguru Nakajima, Yuichiro Otsuka, Yoshitaka Kaneita, Osamu Itani, Yuki Kuwabara, Aya Kinjo, Ruriko Minobe, Hitoshi Maesato, Susumu Higuchi, Hideyuki Kanda, Hisashi Yoshimoto, Maki Jike, Hideaki Kasuga, Teruna Ito, Yoneatsu Osaki

**Affiliations:** 1 Division of Public Health, Department of Social Medicine, Nihon University School of Medicine, 30-1 Oyaguchi Kami-cho, Itabashi-ku 173-8610, Japan; nakajima.suguru@nihon-u.ac.jp (S.N.); kaneita.yoshitaka@nihon-u.ac.jp (Y.K.); itani@iuhw.ac.jp (O.I.); 2 Division of Environmental and Preventive Medicine, Department of Social Medicine, Faculty of Medicine, Tottori University, 86 Nishi-cho, Yonago 683-8503, Japan; ykuwabara@tottori-u.ac.jp (Y.K.); kinjo_aya@tottori-u.ac.jp (A.K.); yoneatsu@tottori-u.ac.jp (Y.O.); 3 National Hospital Organization Kurihama Medical and Addiction Center, 5-3-1 Nobi, Yokosuka 239-0841, Japan; r15candy@yahoo.co.jp (R.M.); h-susumu@db3.so-net.ne.jp (S.H.); 4 National Hospital Organization, Ryukyu Hospital, 7958-1 Kin, Kunigami 904-1201, Japan; jintan@chive.ocn.ne.jp; 5 Department of Public Health, Okayama University Graduate School of Medicine, Dentistry and Pharmaceutical Sciences, 1-1-1 Tsuchimanaka, Kita-ku 700-0082, Japan; hkanda@okayama-u.ac.jp; 6 Department of Family Medicine, General Practice and Community Health, Faculty of Medicine, University of Tsukuba, 1-1-1 Tennodai, Tsukuba 305-8577, Japan; hisashiyoshi@md.tsukuba.ac.jp; 7 Department of Food Science and Nutrition, Faculty of Life and Environmental Science, Showa Women’s University, 1-7-57 Taishido, Setagaya-ku 154-8533, Japan; m-jike@swu.ac.jp; 8 Department of Hygiene and Preventive Medicine, Fukushima Medical University, 1 Hikarigaoka, Fukushima 960-1247, Japan; h-kasuga@fmu.ac.jp; 9 Department of Food and Nutrition, Koriyama Women’s University, 25-2 Kaisei 3-chome, Koriyama 963-8503, Japan; teruna.ito@koriyama-kgc.ac.jp

**Keywords:** alcohol drinking, female, sleep duration, sleep quality

## Abstract

It is unclear whether patterns of alcohol consumption are associated with sleep disturbance. We aimed to investigate the relationship between comprehensive alcohol-related factors and insomnia symptoms, as well as sleep quality, among adult women in Japan. Responses to an online cross-sectional survey were gathered from 12,000 women. The survey items included demographic characteristics, alcohol consumption (Alcohol Use Disorders Identification Test, nightcaps, years of drinking), sleep-related factors (sleep duration, insomnia symptoms, sleep quality), lifestyle-related factors, and mental health. Binary logistic regression was used to investigate the relationship between alcohol consumption and both insomnia symptoms and sleep quality. A total of 10,233 women were included in the final analysis. The results revealed that several alcohol-related behaviors, including the consumption of nightcaps and years of drinking, were significantly associated with insomnia symptoms and poor sleep quality. Specifically, nightcaps were significantly associated with all types of insomnia symptoms and poor sleep quality, with a higher odds ratio than other alcohol-related items. Our findings suggest that specific alcohol-related behaviors, particularly the consumption of nightcaps, are associated with insomnia symptoms and poor sleep quality among women. Intervention programs for alcohol consumption should be provided to prevent sleep problems among women.

## 1. Introduction

Alcoholic beverages are sold in different varieties and are enjoyed widely. However, their accessibility increases the risk of problematic drinking behaviors among individuals who struggle to control their intake. There are various types of problem drinking, ranging from excessive alcohol consumption to alcohol-induced memory impairment and dependency [1]. Problem drinking can cause illness and injury to both the drinker and individuals surrounding them, who account for 5.1% of the total disease and injury burden [2]. In addition, problem drinking imposes considerable social and economic costs and is a significant public health issue [2].

Sleep-related problems are among the health effects of problem drinking [3], and include insomnia symptoms (difficulty initiating sleep (DIS), difficulty maintaining sleep (DMS), early morning awakening (EMA)) and poor sleep quality. These symptoms often occur as early-onset symptoms of various psychiatric disorders, including depression, and can increase the risk of mental illness [4]. Additionally, chronic sleep disturbances are associated with an increased risk of obesity, diabetes, and heart disease [5,6]. Therefore, good sleep contributes to maintaining physical and mental health.

Problem drinking is associated with insomnia, with ≥40% of people diagnosed with alcohol use disorder (AUD) also reporting insomnia symptoms [7]. Notably, insomnia has been associated with an increased risk of problem drinking resulting from self-medication with alcohol for its soporific effects [8]. In Japan, 30.3% of individuals consume alcohol at bedtime as a sleeping aid, which is a considerably higher percentage than in other countries [9]. Japanese drinking culture is deeply embedded in social and professional spheres, serving multiple functions. It operates as a “third space” between work and the home, facilitating relaxation and an escape from hierarchical constraints while fostering harmonious relationships [10]. University sports clubs play a significant role in educating students about alcohol consumption, preparing them for future social interactions [11]. Various alcoholic beverages, including sake, shochu, and beer, are consumed in diverse social settings such as izakaya, snack bars, and private residences [12]. Alcohol consumption is frequently associated with work-related activities and stress alleviation [12], contributing to frequent drinking, which is thought to affect sleep.

Although numerous studies have described associations between alcohol consumption and sleep, most participants have been men, with few studies describing these relationships in women. This could be because, compared with women, men are at a higher risk of developing AUD and alcohol-related death [13]. However, several small-scale studies in female populations have reported similar associations between alcohol consumption and sleep quality to those observed in men [14]. Notably, data from the 2019 National Health and Nutrition Survey revealed that 9.1% of Japanese women consume alcohol in amounts that are likely to increase their risk of lifestyle diseases, reflecting a significant increase since 2010 [15].

Most studies on the relationship between alcohol and sleep have focused on the amount and frequency of alcohol consumption. However, studies on physical illnesses and injuries have revealed that drinking patterns, not only the amount and frequency of alcohol consumption, are also associated with these illnesses [16]. To our knowledge, it remains unclear whether patterns of alcohol consumption are associated not only with physical illness and injury but also with sleep disturbances. Identifying these associations could lead to more specific methods for the moderation of alcohol intake and preventative education. This study hypothesized that alcohol consumption patterns are associated with insomnia symptoms. Accordingly, we aimed to examine the relationship between various alcohol-related factors, insomnia symptoms, and sleep quality in Japanese adult women.

## 2. Results

Among the 12,000 participants, we excluded 1767 because of missing weight data or other information, resulting in the final sample of 10,233 participants.

### 2.1. Participant Characteristics

#### 2.1.1. Demographic Characteristics

Table 1 presents demographic characteristics. Among the participants, 10.7% met the criteria for obesity, 8.3% smoked cigarettes (0.6% heavy smokers, 7.7% light smokers), 6.0% used hypnotic medication more than once a week, 8.4% reported severe mental health impairment, and 28.4% reported moderate mental health impairment.

#### 2.1.2. Alcohol-Related Factors

Table 2 presents the characteristics related to alcohol consumption. Among the participants, the most common factors, in order of prevalence, were a drinking history of ≥10 years, an alcohol consumption frequency of >3 days per week, a typical alcohol consumption of more than two drinks, an increased priority given to drinking, problem drinking, guilt after drinking, the consumption of nightcaps, alcohol-induced blackouts, impaired control over drinking, heavy occasional drinking, morning drinking, concerns from others about drinking, and alcohol-related injuries.

Excessive drinking means that the typical quantity of alcohol consumption is >2 drinks.

#### 2.1.3. Sleep-Related Factors

Table 3 presents details of the participants’ sleep patterns. Among the participants, a short sleep duration, poor sleep quality, DIS, DMS, and EMA were the most common, in that order.

### 2.2. Associations Between Alcohol Consumption and Sleep Quality

**Figure 1:**
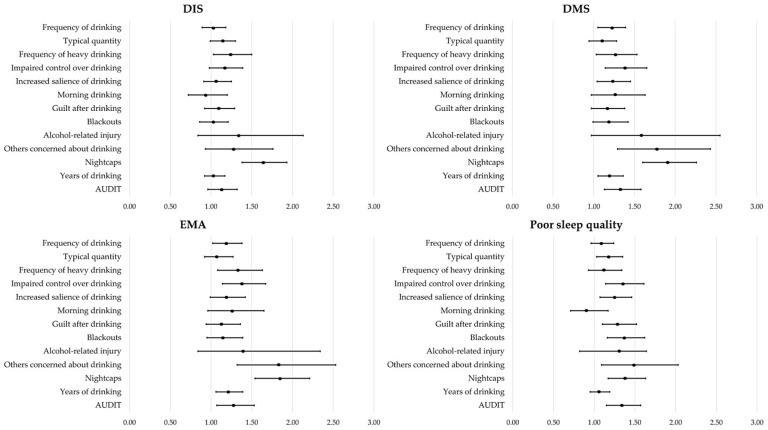
Association of alcohol use patterns with insomnia symptoms and poor sleep quality. DIS, difficulty initiating sleep; DMS, difficulty maintaining sleep; EMA, early morning awakening; CI, confidence interval; AUDIT, Alcohol Use Disorders Identification Test.

Figure 1 describes the associations between various alcohol consumption behaviors and insomnia symptoms, as well as sleep quality.

The frequency of excessive drinking (adjusted odds ratio (AOR): 1.24, 95% confidence interval (CI): 1.03–1.50, *p* = 0.020) and the consumption of nightcaps (AOR: 1.64, 95% CI: 1.38–1.93, *p* < 0.001) were significantly associated with DIS.

The frequency of alcohol consumption (AOR: 1.21, 95% CI: 1.05–1.39, *p* = 0.009), the frequency of excessive drinking (AOR: 1.26, 95% CI: 1.03–1.53, *p* = 0.023), impaired control over drinking (AOR: 1.37, 95% CI: 1.14–1.65, *p* = 0.001), an increased priority given to drinking (AOR: 1.23, 95% CI: 1.04–1.45, *p* = 0.018), concerns from others about drinking (AOR: 1.77, 95% CI: 1.29–2.43, *p* < 0.001), the consumption of nightcaps (AOR: 1.90, 95% CI: 1.60–2.26, *p* < 0.001), the years of drinking (AOR: 1.19, 95% CI: 1.05–1.36, *p* = 0.008), and the Alcohol Use Disorders Identification Test (AUDIT) (AOR: 1.33, 95% CI: 1.13–1.58, *p* = 0.001) were significantly associated with DMS.

The frequency of alcohol consumption (AOR: 1.19, 95% CI: 1.02–1.38, *p* = 0.026), the frequency of excessive drinking (AOR: 1.33, 95% CI: 1.08–1.63, *p* = 0.007), impaired control over drinking (AOR: 1.38, 95% CI: 1.14–1.67, *p* = 0.001), concerns from others about drinking (AOR: 1.83, 95% CI: 1.32–2.53, *p* < 0.001), the consumption of nightcaps (AOR: 1.85, 95% CI: 1.54–2.21, *p* < 0.001), the years of drinking (AOR: 1.21, 95% CI: 1.06–1.39, *p* = 0.006), and the AUDIT (AOR: 1.28, 95% CI: 1.07–1.53, *p* = 0.007) were significantly associated with EMA.

The typical quantity (AOR: 1.18, 95% CI: 1.03–1.35, *p* = 0.015), impaired control over drinking (AOR: 1.35, 95% CI: 1.14–1.61, *p* < 0.001), an increased priority given to drinking (AOR: 1.25, 95% CI: 1.07–1.46, *p* = 0.005), guilt after drinking (AOR: 1.29, 95% CI: 1.10–1.52, *p* = 0.002), blackouts (AOR: 1.37, 95% CI: 1.16–1.62, *p* < 0.001), concerns from others about drinking (AOR: 1.49, 95% CI: 1.09–2.03, *p* = 0.013), the consumption of nightcaps (AOR: 1.38, 95% CI: 1.17–1.63, *p* < 0.001), and the AUDIT (AOR: 1.34, 95% CI: 1.15–1.57, *p* < 0.001) were significantly associated with poor sleep quality.

## 3. Discussion

Our findings indicate that insomnia symptoms and sleep quality are significantly associated with several alcohol-related behaviors, including the consumption of nightcaps and years of drinking. Notably, among all alcohol-related behaviors, the consumption of nightcaps demonstrated the strongest association with insomnia symptoms and overall sleep quality.

Our findings showed that nightcaps were linked to all insomnia symptoms and poor sleep quality, contrary to reports suggesting that they aid in sleep initiation [17]. This discrepancy may stem from alcohol tolerance. While alcohol initially shortens the sleep onset latency due to sedation, this effect diminishes within 3–7 days of regular use [18], rendering chronic nightcaps ineffective for inducing sleep. Since we did not examine the timing of the respondents’ nightcaps, it remains unclear whether they were consumed immediately before bed. However, metabolic arousal occurring hours later, as the blood alcohol levels approach zero, may disrupt sleep initiation [18,19]. Some individuals may also use nightcaps to self-medicate for pre-existing sleep issues, but as their tolerance develops, DIS may recur [8]. Our findings that nightcaps are associated with DMS and EMA are consistent with prior studies showing that alcohol before bed suppresses REM sleep, leading to lighter sleep and awakenings later in the night [7,18]. The observed link between the consumption of nightcaps and poor sleep quality suggests that nightcaps, intended to improve sleep, may instead worsen insomnia symptoms.

An alcohol consumption frequency of >3 days per week was associated with DMS and EMA, consistent with previous research [20]. This could be attributed to metabolic arousal caused by the processes involved in alcohol metabolism [7,18]. Specifically, other than before bedtime, alcohol is most commonly consumed during the evening meal; nonetheless, alcohol can interfere with sleep long after consumption [18,19]. Drinking more than three days a week was not associated with poor sleep quality. Further, unlike taking a nightcap for the purpose of improved sleep quality, consuming alcohol only for the purpose of enjoyment did not lead to dissatisfaction with sleep quality.

Excessive drinking was associated with poor sleep quality but not insomnia symptoms. The metabolism of alcohol starts immediately after its consumption, with the blood alcohol levels and metabolites, especially acetaldehyde, significantly affecting sleep. When alcohol is consumed close to bedtime, the blood alcohol levels may continue to rise even after sleep onset, impacting sleep for hours until the alcohol is fully metabolized [17,21]. Our findings differ from those of previous studies, possibly because we only measured the amount of alcohol consumed at one time without considering the frequency or timing. Since insomnia symptoms were assessed based on the past month, alcohol may not have influenced a respondent’s sleep if it was not consumed during that period.

Heavy occasional drinking was associated with DIS, DMS, and EMA. Alcohol ingestion at large (≥1 g/kg) and moderate-to-small (<1 g/kg) amounts has different effects on sleep. Multiple studies using overnight polysomnography have reported a reduced duration of REM sleep following alcohol ingestion at low and moderate amounts [3,7]. Contrastingly, consuming large amounts of alcohol increases the sleep onset latency, reduces sleep efficiency, increases the amounts of non-REM and REM sleep in the latter half of the night, increases difficulty in maintaining sleep, and lengthens waking times at night [3,7]. Accordingly, the repeated consumption of large amounts of alcohol may be associated with nighttime awakening.

Impaired control over drinking was associated with DMS, EMA, and poor sleep quality. Similarly, an increased priority given to drinking was linked to DMS and poor sleep quality. Both impaired control and an increased priority are symptoms of alcohol dependency. Individuals struggling to regulate their drinking may adopt an alcohol-centered lifestyle, leading to excessive consumption [22,23]. Additionally, alcohol dependency can result in neglecting social activities and health-related behaviors, such as regular meals and exercise, which help regulate circadian rhythms. This neglect may disrupt circadian rhythms and contribute to circadian rhythm sleep disorder [24,25]. Thus, impaired control and an increased priority given to drinking may be associated with sleep outcomes through causing excessive alcohol intake and circadian rhythm disruptions.

Morning drinking was not associated with insomnia symptoms or poor sleep quality. This could be because the metabolic processing of alcohol consumed in the morning is typically completed by the onset of sleep. However, a study on agricultural workers observed a relationship between morning drinking and insomnia [26]. Since we did not consider differences in the working environment or mental health, future studies are warranted.

Guilt after drinking was linked to poor sleep quality but not insomnia symptoms. Persistent alcohol consumption despite feelings of guilt suggests impaired control over drinking, which might be expected to correlate with insomnia symptoms. However, we did not observe this association. This may be due to the potential motivation to reduce alcohol intake triggered by guilt. Previous studies have shown that individuals who experience guilt after drinking often seek positive changes in themselves [27,28]. Future research should explore the behavioral changes driven by guilt after drinking.

Experiencing alcohol-induced blackouts was associated with poor sleep quality but not insomnia symptoms. Blackouts are characterized by temporary amnesia, wherein individuals are unable to form memories of events after drinking alcohol. Excessive drinking and a rapid rise in the blood alcohol concentration are factors in blackouts [29]. Although alcohol ingestion increases the risk of insomnia symptoms, the amnesic effect of blackouts may prevent individuals from recognizing or recalling their sleep disturbances.

Concerns from others about drinking were associated with DMS, EMA, and poor sleep quality. Such concerns suggest that an individual’s alcohol intake is objectively high. Moreover, people tend to understate their alcohol intake when self-reporting, either due to memory loss or a sense of embarrassment [30]. Accordingly, evaluating concerns from others and the nature of those concerns may be a more appropriate way of elucidating an individual’s drinking habits.

A drinking history of ≥10 years was associated with DMS and EMA. Alcohol has neurotoxic effects and affects receptors that are critical to sleep. Long-term alcohol consumption affects neurons in the cerebral cortex that are necessary for slow-wave sleep, resulting in sleep disorders [14,17]. Thus, 10 years of alcohol consumption may be sufficient to cause neuronal damage.

Consistent with previous studies, nightcaps were significantly associated with DMS and EMA but not with DIS [7,17,18]. Our findings were also consistent with previous research indicating that the frequency of drinking was significantly associated with DMS and EMA [20]. However, unlike previous studies, we did not find a significant association between the typical alcohol consumption volume and DIS, DMS, or EMA [14,21].

Problem drinking was associated with DMS, EMA, and poor sleep quality. Given that problem drinking involves multiple problematic drinking behaviors, its link to insomnia symptoms is expected. More than 40% of individuals with AUDs and significant problem drinking experience insomnia. Considering that various drinking behaviors are associated with insomnia symptoms, identifying the specific behaviors responsible for insomnia is essential [7].

Although our cross-sectional study does not allow causal inferences, certain drinking behaviors, particularly the consumption of nightcaps, may affect sleep patterns. Raising awareness of this possibility through school education and workplace training could help promote responsible drinking. In addition, health checkups and other opportunities should be used to identify individual drinking behaviors and their effects, enabling targeted health guidance to promote well-being.

The present study has several strengths. First, this is the first study to comprehensively evaluate drinking-related factors among women with respect to their relationship with sleep. Second, it involved a sufficiently large sample size with a relatively even age distribution. Third, confounding factors for sleep were extensively adjusted.

However, this study also has some limitations. First, as a cross-sectional study, causal relationships among the variables could not be established. Longitudinal studies are needed to clarify these relationships. Second, relying on self-reported data for alcohol consumption and sleep assessments may have introduced reporting bias. Future studies should use objective measures, such as biosensors for the transdermal alcohol concentration and sleep electroencephalograms. Third, this study was conducted online. Thus, the inability to precisely define the target population and the presence of self-selection bias among respondents constitute significant methodological limitations, potentially resulting in findings that lack generalizability [31]. Nevertheless, the research company that administered the survey has the largest number of registered users in Japan, encompassing 86.2% of Japanese internet users [32]. Fourth, other factors influencing sleep, including other sleep disorders, physical illnesses, socioeconomic factors (income and economic activity), and the use of non-hypnotic medications, were not analyzed. Since insomnia can occur in isolation or alongside comorbidities, it is important to account for physical illnesses and other sleep disorders [33]. In terms of socioeconomic factors, previous studies have reported that insomnia is more pronounced among the unemployed and self-employed [34]. In addition, as the term “drug-induced insomnia” suggests, non-hypnotic medications may impact sleep patterns [35]. Including these unmeasured confounding factors may yield different results, warranting further investigation. Fifth, because this study included only women, sex differences could not be examined. Finally, the cut-off points for certain drinking-related factors were relatively arbitrary. While they were chosen to reflect potential effects on sleep over the past month, alternative cut-offs may produce different findings.

## 4. Materials and Methods

### 4.1. Participants

We conducted an online survey of women registered with an internet-based research company (number of registered users: 2.2 million, Rakuten Insight, Inc., Tokyo, Japan) between 29 September 2021, and 5 October 2021. Among a total of 233,855 registered women aged 20–79 years, e-mail invitations were sent randomly to 43,029 (Supplementary Material S1). Once the number of survey respondents reached the predetermined sample size, invitations were discontinued. Registered respondents were invited to participate via an e-mail, which directed them to the company’s website to complete the survey. Participants were then required to access an electronic informed consent form, agree to participate in the study after reviewing it, and consent to the publication of the study’s results. As compensation, they received shopping points. Based on previous studies which found that the prevalence of alcohol dependency among women was 0.1% [36], the sample size was determined as 12,000 (2500 participants from each of the following age groups: 20–29, 30–39, 40–49, and 50–50 years old, and 1000 participants from each of the following age groups: 60–69 and 70–79 years old). This study was approved by the Ethics Committee of the Faculty of Medicine at Tottori University (Approval No. 22A0007).

### 4.2. Variables

The questionnaire collected data on demographic characteristics (age, height, weight), alcohol-related factors, sleep-related factors, the use of hypnotic medication, and mental health status.

#### 4.2.1. Alcohol-Related Factors

Alcohol-related factors included problem drinking, nightcaps, and years of drinking. Participants were asked if they had ever consumed alcohol, with only those who had experience drinking alcohol completing the items regarding alcohol-related factors. Individuals without experience consuming alcohol were classified as non-drinkers.

Problem drinking was assessed using the AUDIT [1], which has demonstrated reliability and validity, and has a Cronbach’s α of 0.67 in Japan [1,37]. The AUDIT allows assessment of the extent of problem drinking using 10 items: frequency of drinking, typical quantity, frequency of heavy drinking, impaired control over drinking, increased priority given to drinking, morning drinking, guilt after drinking, alcohol-induced blackouts, alcohol-related injuries, and concerns from others about drinking. Since some drinking behaviors lacked clearly defined cut-offs, the cut-offs were defined based on their potential effect on sleep in the past month.

##### Frequency of Drinking

In this study, the frequencies of drinking were more segmented than those described in the AUDIT. Specifically, the following options were provided: “More than twice a day”, “Once a day”, “5–6 days/week”, “3–4 days/week”, “1–2 days/week”, “2–3 days/month”, “1 day/month”, “6–11 days/year”, “1–5 days/year”, and “None in the past year”. Individuals with a drinking frequency of ≥3 days/week were categorized as habitual drinkers [38]. The responses were categorized as “<3 days/week” or “≥3 days/week”. Responses from non-drinkers were categorized as “<3 days/week”.

##### Typical Quantity

Typical quantity was assessed using standard measures of alcohol. Based on Japanese standard measures of alcohol, one drink is equivalent to 10 g of pure alcohol. Participants were shown a drink conversion table for different alcohol types and asked to indicate the number of alcohol drinks they would typically consume (“None”, “1–2 drinks”, “3–4 drinks”, “5–6 drinks”, “7–9 drinks”, or “≥10 drinks”). Two drinks was considered an acceptable alcohol intake [39]. Responses regarding typical quantity were categorized as “Not excessive” or “Excessive”. Responses from non-drinkers were categorized as “Not excessive”.

##### Frequency of Heavy Drinking

We asked about the frequency with which respondents consume ≥6 drinks within a single drinking session (“Never”, “Less than once a month”, “Once a month”, “Once a week”, “Every day or almost every day”). Heavy occasional drinking was defined as the consumption of ≥60 g of pure alcohol on one or more occasions within 30 days [40]. Responses indicating heavy drinking occurring less than once a month or at least once a month were categorized as “Heavy occasional drinking—Absent” or “Heavy occasional drinking—Present”, respectively. Responses from non-drinkers were categorized as “Heavy occasional drinking—Absent”.

##### Impaired Control over Drinking

We asked how often, within the past year, respondents were unable to stop drinking once they started, with options ranging from “Never” to “Every day or almost every day”. Responses indicating the presence or absence of such an experience within the previous year were categorized under “Impaired control” or “No impaired control”, respectively. Responses from non-drinkers were categorized under “No impaired control”.

##### Increased Priority Given to Drinking

We asked how many times within the past year respondents were unable to do something due to drinking that they could have done under normal circumstances, with options ranging from “Never” to “Every day or almost every day.” Responses indicating the presence or absence of such an experience were categorized under “Increased priority” or “No increased priority” given to drinking, respectively. Responses from non-drinkers were categorized under “No increased priority”.

##### Morning Drinking

We asked how often in the past year respondents consumed alcohol in the morning to feel better following a heavy drinking session, with options ranging from “Never” to “Every day or almost every day.” Responses indicating the absence or presence of such an experience were categorized under “No morning drinking” or “Morning drinking”, respectively. Responses from non-drinkers were categorized under “No morning drinking”.

##### Guilt After Drinking

We asked respondents how often in the past year they felt guilty or remorseful after drinking alcohol, with options ranging from “Never” to “Every day or almost every day.” Responses were categorized as “No guilt” (absence of such experiences) or “Guilt” (presence of such experiences). Non-drinkers were included in the “No guilt” category.

##### Blackouts

We asked respondents how often in the past year they experienced memory blackouts after drinking alcohol, with options ranging from “Never” to “Every day or almost every day.” Responses were classified as “No blackouts” (no occurrences in the past year) or “Blackouts” (at least one occurrence). Non-drinkers were included in the “No blackouts” category.

##### Alcohol-Related Injuries

We asked whether the respondent or someone else had ever been injured due to their drinking (“Never,” “Yes, but not in the past year,” or “Yes, in the past year”). Responses were classified as “No injury” (no incidents in the past year) or “Injury” (incidents in the past year). Non-drinkers were included in the “No injury” category.

##### Concerns from Others About Drinking

We asked if a family member, relative, friend, doctor, or other healthcare professional had expressed concern about the respondent’s drinking or had encouraged them to reduce their alcohol consumption (“Never”, “Yes, but not in the past year”, “Yes, in the past year”). Responses indicating the absence or presence of such an experience were categorized as “No” or “Yes”, respectively. Responses from non-drinkers were categorized under “No”.

##### Nightcaps

We asked how often respondents consumed alcohol at night to help them sleep (“Every day”, “5–6 days/week”, “3–4 days/week”, “1–2 days/week”, “2–3 days/month”, “Once a month”, “6–11 days/year”, “1–5 days/year”, “Never in the past year”). Responses were categorized as “No” (less than one nightcap per week) or “Yes” (one or more nightcaps per week) [41]. Non-drinkers were included in the “No” category.

##### Years of Drinking

We asked respondents the number of years they had been consuming alcohol, excluding periods of abstinence. In Japan, heavy drinkers are defined as having ≥10 years of drinking [42]. Responses were grouped as “<10 years” or “≥10 years,” with non-drinkers included in the “<10 years” category.

##### AUDIT

Each of the 10 items from the AUDIT was scored from 0 to 4 points, with the total score being calculated. According to the AUDIT criteria, we categorized a score of ≥8 points as “Problem drinking” and a score of <8 points as “No problem drinking” [37]. Non-drinkers were included in the “No problem drinking” category.

#### 4.2.2. Sleep-Related Factors

##### Insomnia Symptoms

We asked participants to rate the frequency at which they experienced insomnia symptoms (DIS, DMS, and EMA) within the previous month (“Never”, “Seldom”, “Sometimes”, “Often”, “Always”). For each insomnia symptom, responses of “Often” or “Always” were categorized as indicative of the corresponding symptom [43].

##### Sleep Quality

We asked respondents to assess the quality of their sleep over the past month (“Very good”, “Good”, “Normal”, “Bad”, “Very bad”). Responses of “Bad” and “Very bad” were categorized as poor sleep quality [43].

##### Sleep Duration

We asked respondents about their average sleep duration over the past month. Short sleep duration (SSD) was defined as <6 h [43].

##### Use of Hypnotic Medication

We asked respondents to rate how often in the previous month they had used hypnotic medication to help them sleep. Responses were categorized as either “Less than once a week” or “More than once a week” [41].

#### 4.2.3. Covariates

Participants were categorized into the following age groups: 20–29, 30–39, 40–49, 50–59, 60–69, and 70–79 years old.

Body mass index (BMI) was calculated for each participant based on self-reported height and weight. BMI values above 25 kg/m^2^ were categorized as “Obese” [44].

Participants who had never smoked or who had not smoked within the previous month were categorized as “Non-smokers”. Current smokers were asked about the number of cigarettes they consumed in a day. Responses of ≤20 and >20 cigarettes a day were categorized under “Light smokers” and “Heavy smokers”, respectively [45].

Mental health status was assessed using the K6, which is a six-item tool for assessing symptoms of psychological distress such as nervousness and hopelessness [46]. Each item was scored from 0 to 4, yielding a total score between 0 and 24. Total scores of 0–4, 5–12, and ≥13 were categorized as “Light”, “Moderate”, and “Severe” impairment of mental health, respectively [45].

### 4.3. Statistical Analysis

Descriptive statistics were calculated for demographic characteristics, alcohol-related factors, and sleep-related factors. Binomial logistic regression was used to analyze the association between alcohol consumption patterns and insomnia symptoms or sleep quality, with the results being reported as AORs and at 95% CIs. Insomnia symptoms and sleep quality were the dependent variables, while alcohol-related factors such as problem drinking, nightcaps, and years of drinking were the independent variables. Given the possibility of multicollinearity among drinking frequency, frequency of heavy drinking, and frequency of nightcaps, each alcohol-related item was analyzed separately. In addition to the age group, covariates included BMI, sleep duration, smoking, use of hypnotic medication, and mental health status, which have been shown to influence insomnia symptoms and sleep quality [17,18,19,21,41]. All statistical analyses were conducted using SPSS version 28 (IBM, Armonk, NY, USA). Statistical significance was set at *p* < 0.05.

## 5. Conclusions

Specific patterns of alcohol consumption were associated with insomnia symptoms and poor sleep quality in women. Specifically, the consumption of nightcaps showed a strong association with sleep disturbances. Individuals with problematic drinking habits who experience insomnia or poor sleep quality should receive counseling to help moderate their alcohol intake. Additionally, individuals using nightcaps should be informed that this habit may worsen insomnia and their sleep quality. Health recommendations during checkups and preventative education in schools and workplaces should support awareness of these results.

## Figures and Tables

**Table 1:** Participant demographics.

	N	% (95% CI)
Age groups (years)		
20–29	2133	20.8 (20.1–21.6)
30–39	2096	20.5 (19.7–21.3)
40–49	2053	20.1 (19.3–20.8)
50–59	2118	20.7 (19.9–21.5)
60–69	911	8.9 (8.4–9.5)
70–79	922	9.0 (8.5–9.6)
BMI		
Obesity	1096	10.7 (10.1–11.3)
Smoking		
Heavy smoker	60	0.6 (0.5–0.8)
Light smoker	784	7.7 (7.2–8.2)
Non-smoker	9389	91.8 (91.2–92.3)
Use of hypnotic medication		
At least once a week	616	6.0 (5.6–6.5)
Mental health		
Severe	858	8.4 (7.9–8.9)
Moderate	2910	28.4 (27.6–29.3)
Light	6465	63.2 (62.2–64.1)

Obesity was defined as a body mass index (BMI) ≥ 25 kg/m^2^. CI, confidence interval.

**Table 2:** Alcohol drinking patterns among Japanese adult women.

	N	% (95% CI)
Frequency of drinking		
≥3 days/week	2137	20.9 (20.1–21.7)
Typical quantity		
Excessive drinking	1766	17.3 (16.5–18.0)
Frequency of heavy drinking		
Heavy occasional drinking	851	8.3 (7.8–8.9)
Impaired control over drinking		
Yes	901	8.8 (8.3–9.4)
Increased salience of drinking		
Yes	1146	11.2 (10.6–11.8)
Morning drinking		
Yes	421	4.1 (3.7–4.5)
Guilt after drinking		
Yes	1062	10.4 (9.8–11.0)
Blackouts		
Yes	968	9.5 (8.9–10.0)
Alcohol-related injuries		
Yes	103	1.0 (0.8–1.2)
Others concerned about drinking		
Yes	237	2.3 (2.0–2.6)
Nightcaps		
Yes	985	9.6 (9.1–10.2)
Years of drinking		
≥10 years	4853	47.4 (46.5–48.4)
AUDIT		
Problem drinking	1119	10.9 (10.3–11.5)

CI, confidence interval; AUDIT, Alcohol Use Disorders Identification Test.

**Table 3:** Sleep status among Japanese adult women.

	N	% (95%CI)
Short sleep duration	4571	44.7 (43.7–45.6)
DIS	1914	18.7 (18.0–19.5)
DMS	1530	15.0 (14.3–15.6)
EMA	1313	12.8 (12.2–13.5)
Poor sleep quality	2298	22.5 (21.7–23.3)

DIS, difficulty initiating sleep; DMS, difficulty maintaining sleep; EMA, early morning awakening; CI, confidence interval.

## Data Availability

The data that support the findings of this study are available from the corresponding author, Y.O., upon reasonable request.

## Supplementary Materials

The following supporting information can be downloaded at: https://www.mdpi.com/article/10.3390/clockssleep7010005/s1, Table S1: The number of registrants of research company and invitations.
