# Supplementary material for: Association Between Alcohol Use Patterns and Insomnia Symptoms or Poor Sleep Quality Among Adult Women: An Internet Cross-Sectional Survey in Japan
Source: Clocks Sleep. 2025 Feb 13;7(1):5. doi: 10.3390/clockssleep7010005 (PMC11843888; doi:10.3390/clockssleep7010005)
Supplement: Supplementary file 1 [file clockssleep-07-00005-s001.zip › clockssleep-3382795-supplementary.pdf]

Supplementary Material S1

Table S1. The number of registrants of research company and invitations

	registrants	invitations
**Age groups (years)**		
**20–29**	34814	12600
**30–39**	62835	8125
**40–49**	58284	8061
**50–59**	46887	7933
**60–69**	23742	3208
**70–79**	7293	3102
**Total**	233855	43029
